# Molecular epidemiology of endemic Human T-Lymphotropic virus 1 (HTLV-1) in a community in rural Guinea-Bissau

**DOI:** 10.1186/1742-4690-8-S1-A71

**Published:** 2011-06-06

**Authors:** Carla van Tienen, Thushan I de Silva, Luiz C Alcantara, Maarten Schim van der Loeff, Tim Vincent, Nato Gonçalves, Roel A Coutinho, Peter Aaby, Hilton Whittle, Matthew Cotten

**Affiliations:** 1 Viral diseases program, Medical Research Council, Fajara, Gambia; 2 Department of Medical Microbiology and Infectious Diseases, Erasmus Medical Centre, Rotterdam, Netherlands; 3 Centre for Medical Molecular Virology, Division of Infection and Immunity, University College London, London, UK; 4 Gonçalo Moniz Research Center, Oswaldo Cruz Foundation, Candeal, Salvador, Bahia, Brasil; 5 Municipal Health Services, Amsterdam, Netherlands; 6 Projecto de Saúde de Bandim, Bissau, Guinea-Bissau; 7 Center for Infection and Immunity Amsterdam, University of Amsterdam, Amsterdam, Netherlands; 8 Projecto de Saúde de Bandim/Indepth Network, Bissau, Guinea-Bissau

## Background

HTLV-1 is endemic in parts of Africa and the highest prevalence (5%) in West Africa has been reported in Caio, a rural area in Guinea-Bissau. It is unknown which HTLV-1 subtypes are present in this community.

## Objective

To compare local HTLV-1 sequence variation and to describe the phylogeny of the virus in the Caio population.

## Methods

The complete Long Terminal Repeat (LTR) region of HTLV-1 samples from children and adults was sequenced. Socio-demographic data were obtained from routine census data and from interviews. Phylogenetic analyses were performed to characterize the viruses.

## Results

The complete 800-bp LTR sequences from 38 HLTV-1 infected adults and 8 children were obtained. Strikingly, sequences from many unrelated individuals showed 100% nucleotide identity (e.g. one group of 10 individuals had identical sequences). Phylogenetic analysis demonstrated that 45 of the sequences belonged to the cosmopolitan subtype A, subgroup D. One sequence, Caio4046, was divergent and formed a significant cluster with the STLV-1 sequences from the Central African Republic and Senegal and with a recently identified HTLV-1 strain from a Cameroonian hunter (tentatively designated subtype G) [1] and belonged to a woman without known monkey contact.

## Conclusions

Markedly conserved HTLV-1 strains of the cosmopolitan subtype A, subgroup D, predominate in this rural community. However, HTLV-1 subtype G is also present. This subtype has not been described before in a non-hunter or in West Africa and suggests this subtype may be more widespread than previously thought. Additional sequencing can give more information about the frequency of this subtype in Caio.
